# Changing medical student attitudes to patient safety: a multicentre study

**DOI:** 10.1186/s12909-018-1313-0

**Published:** 2018-08-28

**Authors:** Kim Oates, Ian Wilson, Wendy Hu, Ben Walker, Amanda Nagle, Janice Wiley

**Affiliations:** 1 NSW Clinical Excellence Commission Rawson Place, Sydney and University of Sydney, Sydney, Australia; 2 Discipline of Child and Adolescent Health, University of Sydney, Sydney, Australia; 3 Faculty of Science, Medicine and Health, School of Medicine, University of Wollongong, Wollongong, Australia; 4 School of Medicine, Western Sydney University, Sydney, Australia; 5 Joint Medical Program, Universities of Newcastle and New England, Newcastle, Australia; 6 School of Rural Medicine, University of New England, Armidale, Australia; 7 School of Medicine, University of Notre Dame, Sydney, Australia

**Keywords:** Educational innovation, Multicentre evaluation, Attitudinal scale, Patient safety, Medical students, Cohort study

## Abstract

**Background:**

Although patient safety is becoming widely taught in medical schools, its effect has been less rigorously evaluated. We describe a multicentre study to evaluate student changes in patient safety attitudes using a standardised instrument, the Attitudes to Patient Safety Questionnaire3 (APSQ3).

**Methods:**

A patient safety training package designed for medical students was delivered in the first year and second year in four Australian medical schools. It comprises eight face-to-face modules, each of two hours. Seminars start with an interactive introduction using questions, video and role play, followed by small group break-outs to discuss a relevant case study. Groups are led by medical school tutors with no prior training in patient safety. Students and tutors then reassemble to give feedback and reinforce key concepts. Knowledge and attitudes to patient safety were measured using the APSQ3, delivered prior to safety teaching, at the end of the first and second years and 12 months after teaching ceased.

**Results:**

A significant improvement in attitude over time was demonstrated for four of nine key items measured by the APSQ3: value of patient safety teaching; danger of long working hours, value of team work and the contribution patients can make in reducing error. Informal feedback from students was very positive.

**Conclusion:**

We showed persistent, positive learning from a patient safety education intervention 12 months after teaching finished. Building on the introduction of patient safety teaching into medical schools, pathways for motivated students such as appropriate electives, option terms and team-based research projects would be of value.

## Background

It cannot be safely assumed that concepts of patient safety will be learned after graduation from medical school. Of those who teach and supervise young doctors, not all will be skilled in and familiar with these concepts. This is one reason why it is now widely accepted that quality and safety education should commence during medical school [1].

In the USA, medical schools are now required to provide basic instruction in patient safety for students and residents in order to be able to meet accreditation requirements [2]. The Australian Medical Council includes teaching quality and safety of healthcare as one of its standards for assessment of medical schools. This is likely to be strengthened in 2018 [3]. In the UK, the General Medical Council works with medical schools to teach all students about patient safety [4].

Concepts of quality and safety in healthcare are becoming recognised as fundamental concepts that are slowly becoming embedded into student teaching in the same way that basic sciences are assumed to be an integral component of the curriculum [1].

Generally however, medical schools do not yet have the depth of skills and experience in patient safety education that they possess in the more traditional areas. For this reason the Association of American Medical Colleges has developed a program aimed at developing a core group of medical educators who are trained and certified in teaching patient safety [5]. Developing such teaching expertise has clear value and is reinforced by a national survey in Australia showing a discrepancy between the amount of patient safety that the deans of medical schools thought was being taught and the amount perceived by students as actually being delivered [6].

Although quality and safety is now widely taught in medical schools, it has been less widely evaluated. Many evaluations have been with small class sizes, conducted in only one medical school, and using a pre-test post-test design with the post-test given immediately at the end of the teaching activity. Not surprisingly, these studies show that knowledge increases [7–11]. One early study, completed by 55 students at a single centre, did assess student knowledge and attitudes one year after they participated in a patient safety course. It showed mixed results with some students showing improvement on the domains measured but others showing no change or change in an undesired direction [12].

A recent systematic review [13] of 26 studies (11 involving medical students), published since January 2009, found that most were from single institutions, with a median sample size of 109. Only two study involving medical students measured effects beyond the immediate post-test, one by conducting a further assessment after six weeks [14]^,^ and one reviewing a small number (17) of students after 6–12 months [15].

This paper describes a multicentre study conducted by the Clinical Excellence Commission (CEC) in Sydney, Australia to evaluate change in patient safety attitudes among medical students. The CEC is a statutory body established in 2004 to improve the quality and safety in health services through training and education initiatives. In 2009 it developed a Patient Safety Training Package for medical students [16]. It now delivers this program in four medical schools.

We aimed to measure the persistence of attitudinal change to patient safety following delivery of the CEC program in four different medical schools. A secondary aim was to validate a patient safety attitudinal scale in a medical student cohort.

One medical school is a four year graduate degree program where a prior degree is an entry prerequisite. The other three are five year undergraduate school leaver entry programs. These three also include some students who have transferred from another course or who already have a degree. Selection criteria differ across these four medical schools, with some using single interview and personal references in addition to academic record while others use multiple mini interviews, plus or minus psychometric testing and do not use references.

## Methods

The study used a descriptive pre-post observational design, non-randomised and non-controlled. The medical education intervention was the CEC Patient Safety Training Package delivered in the first year and second years of the medical curriculum. It consists of four face-to-face 2-h modules delivered as a seminar + tutorial, throughout first year and another four delivered throughout second year, a total of 8 modules each of two hours. Each seminar starts with a 40-min interactive introduction to the topic, where role play and video clips were used as well as question and answer. This is delivered at all sites by the same academic teacher from the CEC. The introduction is followed by small group break-outs where a relevant case study is discussed. All students in first and second year of the 4 medical schools were eligible to participate in the study.

Each group is led by a tutor from the host medical school who is often also the students’ Problem Based Learning (PBL) tutor. Tutors are predominantly medical graduates, with some from allied health, nursing and medical science. None have had prior specific training in patient safety. Students participate in these tutor lead discussions for 50–60 min and then reassemble as a cohort to give feedback from the tutorial, with key concepts being reinforced. Some of the curriculum material was adapted from the IHI Open School [17] and the WHO Patient Safety Curriculum [18].

The first four modules of the CEC Patient Safety Training Package (delivered to Year 1) cover:

Why errors occur;

Blame and safe cultures;

How students can use leadership skills to make patients safer;

The importance of listening to patients and families.

The second four, delivered when these students were in their second year, cover:

Human factors;

Teams and communication;

Open disclosure;

Involving patients as part of the care team.

In addition to having students think through the issues highlighted by the case study, the teaching sessions are designed to enhance the understanding of patient safety concepts amongst the tutors from the relevant medical school who attend the introductory component of each session prior to leading a break-out discussion group. This may have potential for the continuing application of these concepts in the PBL sessions led by those same tutors throughout the year.

Knowledge and attitudes to patient safety were measured using the Attitudes to Patient Safety Questionnaire version 3 (APSQ 3). This measure comprises 26 items (statements) across nine key patient safety factors [19]. Each item is scored on a Likert type scale with higher scores indicating more positive attitudes to patient safety, although items that show negative attitudes to patient safety concepts are reverse scored [19].

Typical statements on the APSQ3 are: “All medical errors should be reported”; “Teaching teamwork skills will reduce medical errors”; “Encouraging patients to be more involved in their care can help reduce the risk of medical error”; “By not taking regular breaks, doctors are at increased risk of making errors”. Statements that are reverse scored include “Medical errors are a sign of incompetence”; “Most medical errors result from careless nurses”.

The APSQ3 was administered to students at the four medical schools immediately before patient safety teaching commenced (Time 1), at the end of first year, when the first four modules had been completed (Time 2), at the end of second year, when the second four modules had been completed (Time 3) and 12 months after patient safety teaching had ceased (Time 4). All surveys were in hard copy and were completed in the class-room in the presence of academic staff, but not in the presence of any of the investigators.

One author who was not involved in any of the medical schools taking part in this study created linked, but de-identified tables of data across the four time points. As such, the data were analysed as linked data. Reverse scored items were re-scored.

In addition to the 26 items on the APSQ3, students provided anonymous information about: their age; the medical school they attended; the name of any prior degree; whether they had any prior experience in any health professional area; whether they had any prior experience in industry.

Human research ethics committee approval was granted by each of the four participating medical schools: University of Notre Dame [ID No 0130275], University of Western Sydney [ID No H9802], University of Newcastle [ID No H-2013-0132], University of New England [ID No H2013–0132]. Informed written consent was obtained from all participants.

The nine key measures of the APSQ3 are described by means and standard deviations. Analysis of the changes was by repeated measures ANOVA with exploration of within and between subject measures.

## Results

### Number of participants

The number of participants from each medical school at each of the four time points is shown in Table 1.

**Table 1:** Number of participants from each medical school at four time points

School	Time 1	Time 2	Time 3	Time 4
A	97	77	68	37
B	77	98	53	1
C	67	70	69	62
D	122	98	87	55
Total	363	343	277	155

 One school (B) had difficulty identifying which of its students completed the survey at Time 4. As all student responses were de-identified, we were only able to confirm one student from this school who had completed the earlier surveys. Ninety nine students completed all four surveys. Only these students were included in the analysis.

Factor scores at the different time points are detailed in Table 2.

**Table 2:** Factor scores at the four time points

		Factor mean score (sd)			
Factor	Title	Time 1	Time 2	Time 3	Time 4
1	Patient safety training received	10.75 (1.28)	12.09 (1.22)	12.33 (1.14)	11.98 (1.13)
2	Error reporting confidence	10.04 (2.02)	10.31 (2.16)	10.42 (2.01)	9.91 (2.11)
3	Working hours as error cause	11.79 (1.49)	12.40 (1.84)	12.41 (1.69)	12.67 (1.54)
4	Error inevitability	12.88 (1.39)	12.99 (1.53)	13.32 (1.34)	13.06 (1.34)
5	Professional incompetence as error cause	10.71 (1.23)	10.66 (1.30)	10.59 (1.29)	10.46 (1.30)
6	Disclosure responsibility	10.27 (1.31)	10.39 (1.41)	10.40 (1.33)	10.17 (1.36)
7	Team functioning	8.30 (0.80)	8.53 (1.12)	8.86 (0.90)	8.62 (0.89)
8	Patient involvement in reducing error	7.62 (1.24)	8.26 (1.05)	8.92 (0.97)	8.15 (1.11)
9	Importance of patient safety in the curriculum	12.13 (1.31)	11.95 (1.54)	12.51 (1.33)	12.43 (1.44)

Factor mean scores across time

(Notes Factors 1, 2, 3, 4, 6, and 9 have a possible range of 3–15. Factors 7 and 8 have a range of 2–10. Factor 5 has a range of 4–20)

### Measurement of change

**Fig. 1:**
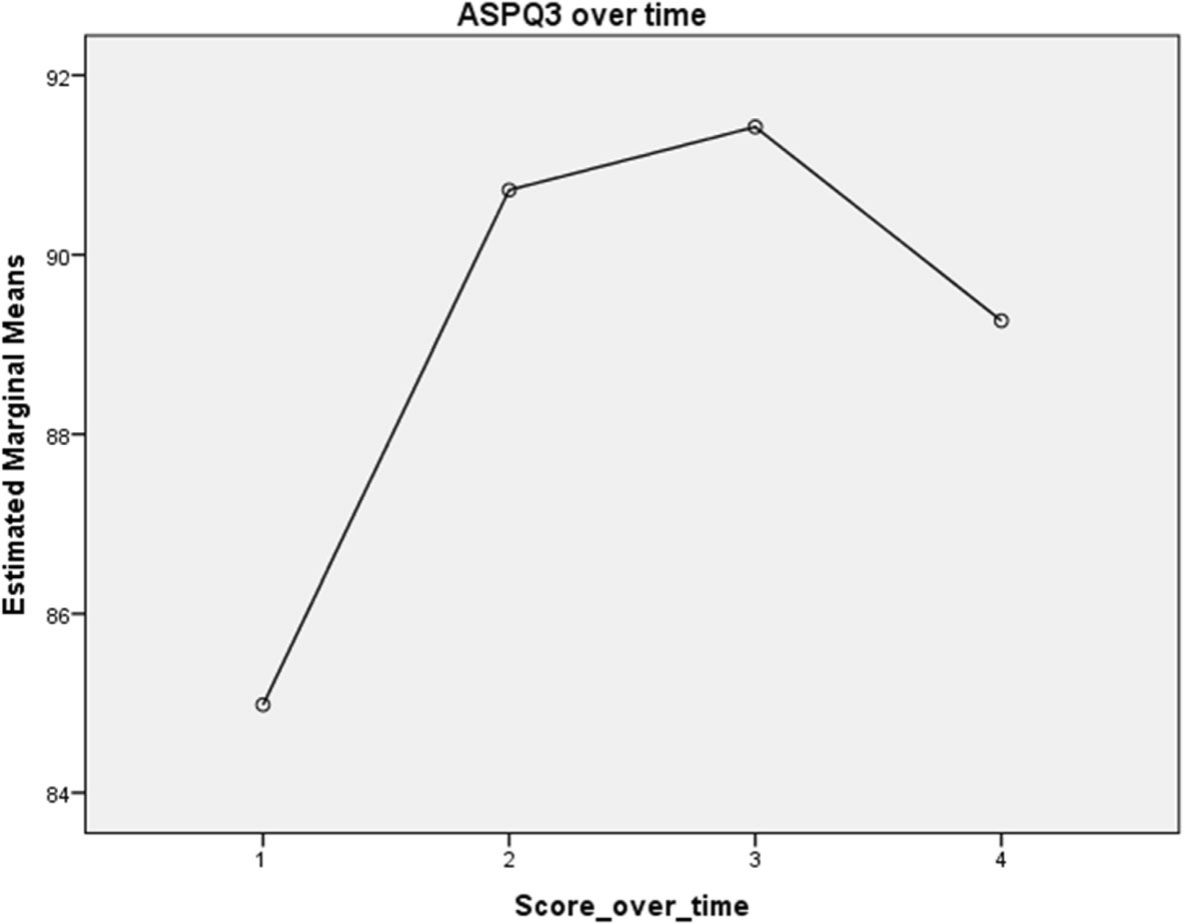
Changes in APSQ3 scores over four time periods

The repeated measures ANOVA with Huyn-Feldt correction determined that, across the four time-points, the APSQ3 scores increased significantly and then declined at the fourth time point (See Fig. 1). Scores at Time1 were significantly lower than scores at other time points, and scores at Time 4 were significantly lower than that at Time3.

Partial Eta Squared indicated that the program explained 28% of the change over time.

Inclusion of between subject factors (university, gender, prior experience of health care, method of entry to medicine and language spoken at home) either individually or together had no impact on the outcome.

A t-test was used for gender, prior experience in a health profession and whether English was the only language spoken at home.. Item 7 (working in teams can reduce errors) showed greater positive change for students who only spoke English at home (.611 vs .143, *p* = .036). Prior experience in a health profession showed an impact on item 4 (errors are inevitable), [prior experience 1.00 vs no prior experience 0.11, *p* = .036] although the number of students with prior experience completing all 4 surveys was only 11. Gender also had an impact on item 4 with the mean difference increasing for male students (.54) and decreasing for females (−.12) (*p* = .01).

These results demonstrate a significant improvement in attitude over time for 4 of the nine key items:

- Item 1, The patient safety training received gives a good understanding of error cause and prevention;
- Item 3, Long working hours and lack of regular breaks can be a cause of errors;
- Item 7, Working in teams can reduce errors;
- Item 8, Patients can play an important role in reducing error.

Informal feedback from these sessions showed that students appreciated them, such as by staying back to ask questions, sending follow up emails, asking for help in initiating patient safety projects or presentations at team meetings and sending vignettes from their own experience and reading that could be used in future teaching sessions.

## Discussion

This study was able to show persistent effects from a patient safety intervention on medical student attitudes across four medical schools.. The intervention comprised eight modules of seminar + tutorial (2 h each) delivered across two years in four medical schools in Australia. Pre-post evaluations, using the APSQ3 of medical student attitudes to patient safety were conducted at baseline (prior to the intervention Time 1), at the end of the first year of medical school (Time 2), at the end of the second year (Time 3) and 12 months after formal patient safety teaching ceased (Time 4). The main differences between the medical schools were their admission criteria and the different tutors who led the discussion groups.

Ninety nine students completed the survey at all four time points. This study has a longer time period between cessation of teaching and safety attitude evaluation than any other study, [12, 20, 21] with only two other studies involving review up to 12 months [15, 22]. In one of these, student numbers were small (38) with the 12 month follow up showing a sustained knowledge increase [22]. In the other study, 17 students were reviewed between six and twelve months after teaching ceased, also showing a sustained improvement [15]. This is the only study we have found that has tested outcomes from a patient safety intervention on students from different medical schools using a common validated instrument, adding robustness to our finding of a persistent effect.

We found that the change in attitude to patient safety on four of the nine key patient safety items measured by the APSQ 3 (Item 1, The patient safety training received give a good understanding of error cause and prevention; Item 3, Long working hours and lack of regular breaks can be a cause of errors; Item 7, Working in teams can reduce errors and Item 8, Patients can play an important role in reducing error) was sustained at a significant level 12 months after teaching ceased. The final post intervention measurement at Time 4 occurred well into the time when students had commenced their clinical placements, a time when erosion of learning in quality and safety may occur. This is particularly the case if students are exposed to “the hidden curriculum” from experience in systems where safety is not seen as a priority or where safety teaching is of poor quality and seen as low status [23, 24].

It is also possible that some other safety teaching may have occurred informally in the 12 months after the CEC program ceased, although until the introduction of the CEC curriculum there had been no formal teaching in any of the curricula of the four medical schools.

Although the four medical schools had different entry and selection criteria to enter the program, as well as different curricula, there were no differences in their APSQ scores at Time 4. This may be a limitation of the study as the consistency of the results may reflect the consistency of the teaching, with the same course delivered by the same teacher. However, only about one third of each seminar was delivered by the same teacher. The majority of the seminars were spent in student discussion groups with tutors from their own medical schools, although all tutors used the same discussion guides for the case studies.

The fact that language spoken by the students at home, student gender and prior experience in health before entering medical school were not strongly associated with differences in results suggests that this type of patient safety teaching is widely applicable.

The most obvious limitation is the lack of a control group. Ideally, attitudes in students not exposed to patient safety teaching could have been used as a comparator. This would have helped resolve the question about whether knowledge just increased over time as part of general clinical exposure. However, as patient safety is now widely taught to medical students in a variety of ways it was not feasible or acceptable to find a control group with no exposure, or even a delayed exposure.

A further limitation is that not all students completed the instrument at all time periods. This was for a variety of reasons, the commonest being absence from a particular session. Future studies using larger samples, minimising loss to follow up, reassessing participants in their early post graduate years and assessing the impact of patient safety teaching on career choices would help demonstrate the likelihood of any longer term impact. In particular, it could be of use to look at why only four of the nine APSQ3 factors showed a sustained improvement and whether more focus in future teaching should be placed on the other five factors where there was no significant sustained change. However, in our study, the number of students who completed the survey at all four time points is almost 6 times greater than the 17 students who were enrolled in the only other published study which assessed student learning beyond the immediate post-test period [15].

Building on the incorporation of patient safety teaching into medical school curricula, there would be value in creating pathways for interested students to explore these areas, learn more and possibly develop careers in this field. Medical schools should be encouraged to offer elective terms, option terms or research projects for students interested in patient safety.

After graduation, opportunities are needed for creating resident medical officer rotating positions in patient safety and quality improvement. The availability of fellowships for those who want to go further will help develop future patient safety clinician-leaders. Initiatives such as these are already starting to occur [25, 26] and are supported by changes in medical graduate competency frameworks around the world, such as CanMEDS [27] and the core professional activities of the Association of American Medical Colleges [28].

## Conclusions

All future medical doctors need to know the principles of patient safety and quality improvement. Our study provides evidence for the potential of a scalable teaching intervention delivered by a centralised body (the CEC in this case), rather than each school developing its own. We expect that such interventions will become more widespread as this area comes to be seen as an integral part of health care for all health professionals as well as a career option for some future leaders.

## Abbreviations

APSQ: Attitudes to patient Safety Questionnaire
CEC: Clinical Excellence Commission
